# Using self-determination theory to predict self-management and HRQoL in moderate-to-severe COPD

**DOI:** 10.1080/21642850.2021.1938073

**Published:** 2021-06-06

**Authors:** Liam Knox, Gareth Norris, Keir Lewis, Rachel Rahman

**Affiliations:** a Department of Psychology, Aberystwyth University, Wales; b Research and Development Department, Hywel Dda University Health Board, Wales; c Medical School, Swansea University, Wales

**Keywords:** Self-determination theory, Chronic Obstructive Pulmonary Disease, Self-management, Health-related quality of life

## Abstract

**Objective:** Chronic Obstructive Pulmonary Disease (COPD) is a long-term condition that detrimentally affects health-related quality of life (HRQoL), with self-management proposed as an effective treatment. Using self-determination theory (SDT), this research explored psychological need satisfaction, frustration, and behavioural regulation to explain indicators of self-management.

**Design and Main Outcome Measures:** Cross-sectional, questionnaire-based methods in people on a pulmonary rehabilitation waiting-list. 72 participants completed SDT, HRQoL, and self-management knowledge questionnaires. Path analyses investigated the ability of SDT concepts to predict self-management knowledge and HRQoL.

**Results:** Chi-square tests found no significant differences (χ^2^(13, N=72) = 16.7, *p *> 0.05) between the just – and over-identified models, and multiple measures suggested an acceptable fit to the data. Relatedness frustration positively predicted controlled regulation and autonomy and relatedness satisfaction positively predicted autonomous regulation. The associations between the other needs and the different regulation types were not statistically significant. Both regulation types strongly predicted HRQoL (35% variance explained) and self-management knowledge (22% variance explained).

**Conclusion:** SDT concepts can predict more self-determined self-management regulation, self-management knowledge, and HRQoL and provide a framework for researchers and healthcare professionals to develop future health interventions for people with COPD. Greater research is needed to understand basic psychological need frustration in health contexts.

## Main text

Chronic Obstructive Pulmonary Disease (COPD) is a progressive, irreversible condition which affects approximately 1.2 million people in the United Kingdom (UK; National Institute for Health and Clinical Excellence [NICE], 2018) and is the third most common cause of death worldwide (Singh et al., 2019). Common symptoms include dyspnoea (breathlessness), sputum production, and coughing (NICE, 2018). Symptoms can get worse during an acute exacerbation – defined as the sudden and prolonged increase in symptoms outside of normal daily fluctuations (NICE, 2018) – which occur on average 1–2 times per year (Dhamane et al., 2015). Symptoms can cause a range of daily living limitations, such as reduced physical ability and feelings of social isolation (Russell et al., 2018). Relative to other chronic conditions, such as stroke, cancer, diabetes mellitus, and coronary artery disease, rates of anxiety and depression are higher in people with COPD, evident in approximately 40% of this population (Khan & Patil, 2017; Phan et al., 2019; Schane, Walter, Dinno, Covinsky, & Woodruff, 2008). Multiple studies have linked depression in people with COPD to a loss of functional mobility and ability to tend to daily tasks, alongside increased symptom burden and mortality (McCathie, Spence, & Tate, 2002; Ng et al., 2007). Therefore, symptoms of COPD may cause anxiety and depression (Schane et al., 2008), which can lead to heightened perceptions of symptoms (Reardon, Lareau, & ZuWallack, 2006). Thus, it is unsurprising that the negative cyclical effect of COPD results in reduced health-related quality of life (HRQoL; Russell et al., 2018). Due to the progressive nature of the disease, people with COPD are extensive healthcare users, representing the second most common cause of emergency hospital admission in the UK and directly costing the National Health Service (NHS) £1847 million in 2014 (Trueman, Woodcock, & Hancock, 2017).

Despite being high users of the NHS, people with COPD spend the vast majority of the time self–managing their condition and symptoms, with only approximately 1% being managed alongside healthcare professionals (NHS, 2014). Relevant COPD self-management behaviours include taking regular exercise and medication, attending healthcare appointments, and smoking cessation (Disler, Gallagher, & Davidson, 2012; NICE, 2018), which aim to maintain an individual’s ability to conduct daily activities and control symptoms (Effing et al., 2016; Singh et al., 2019). Effective self-management has been shown to decrease hospitalisations (Zwerink et al., 2014) and increase wellbeing, HRQoL, and multiple disease-specific outcomes (Benzo, Abascal-Bolado, & Dulohery, 2016; Majothi et al., 2015; Warwick, Gallagher, Chenoweth, & Stein-Parbury, 2010; Zwerink et al., 2014). Supporting self-management behaviours and HRQoL have therefore been identified as an important feature of the care offered to people with COPD (Kielmann et al., 2010).

Despite the benefits of effective self-management, these behaviours are often neglected by people with COPD (Hillebregt, Vlonk, Bruijnzeels, van Schayck, & Chavannes, 2016; Korpershoek et al., 2016; Russell et al., 2018). This is illustrated by examining the attendance and adherence rates for Pulmonary Rehabilitation (PR); a multidisciplinary programme for people with moderate-to-severe respiratory conditions, generally consisting of physical exercise, self-management training, and psychological and smoking cessation support (NICE, 2018; Wagg, 2012). Even though PR has been shown to be a very effective and well-received treatment (McCarthy et al., 2015; Rapport et al., 2014; Singh et al., 2019), of the people referred to the programme, only 69% attend the initial assessment and 42% complete the course (Steiner et al., 2015, 2016).

Studies have begun to investigate why people with COPD do not engage in self-management activities, finding a range of factors including low motivation and a lack of emphasis on behavioural change (Hillebregt et al., 2016; Russell et al., 2018; Sheldon, Williams, & Joiner, 2003). Although identifying these barriers is beneficial in the endeavour to increase self-management behaviours, to fully conceptualise determinants and facilitate the development of interventions, psychological theory should be utilised (Colquhoun, Squires, Kolehmainen, Fraser, & Grimshaw, 2017; Craig et al., 2008). Unfortunately, even though research has found the use of psychological theory positively improves healthcare interventions in a variety of conditions (Michie, Rothman, & Sheeran, 2007; Painter, Borba, Hynes, Mays, & Glanz, 2008), reviews have reported very few primary studies utilise such models (Davies, Walker, & Grimshaw, 2010; Painter et al., 2008; Prestwich et al., 2014). This atheoretical tendency is even more prevalent in studies with populations with COPD. Richardson et al. (2014) performed a review of self-management interventions in chronic disease, finding that only 25% of included COPD studies used a psychological theory. Similarly, a review investigating the use of behaviour change theory for interventions to enhance adherence in chronic respiratory disease found none of the included COPD studies had a theoretical underpinning (McCullough et al., 2016). Given that successful self-management requires a change of, and adherence to, a wide range of new behaviours and lifestyles (Effing et al., 2016) – which is rarely accomplished by a significant number of people with COPD (Russell et al., 2018) – the lack of theory is restricting the current understanding of this important topic and limiting beneficial solutions that could enhance care.

### Self-determination theory

One psychological theory that has demonstrated successful outcomes in other chronic conditions is Self-Determination Theory (SDT; Deci & Ryan, 1985; Hagger & Chatzisarantis, 2015; Ryan & Deci, 2017). This theory describes the mechanisms by which psychological needs influence behavioural regulation and provides clear guidelines for interventions to effectively change the environment, in support of improved need satisfaction and self-determined regulation towards behaviour change.

**Figure 1.**
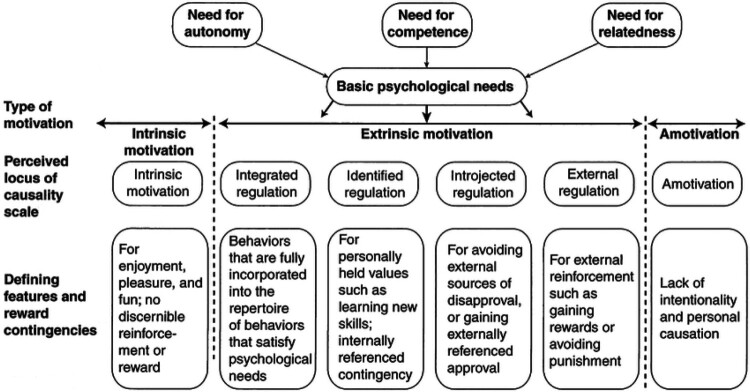
The basic psychological needs and self-determined motivation. Adapted from Hagger and Chatzisarantis (2007).

SDT (presented in Figure 1) focuses on the *quality* of an individual’s motivation, as opposed to other psychological models which emphasise the *quantity* of motivation (Sheldon et al., 2003). The theory posits that there are three basic psychological needs; autonomy, competence, and relatedness (Ryan & Deci, 2017). Autonomy is the need to be a causal factor in one’s life and engage in actions due to interest and integrated values (Ryan & Deci, 2017). Competence refers to the need to feel environmental effectiveness and have opportunities to demonstrate and enact on one’s capabilities (Deci, 1975). Lastly, relatedness is the need to feel connected to other individuals or groups and to care, and be cared for, by these significant others (Ryan, 1995). The more that these needs are satisfied, the greater an individual’s self-determined motivation will be toward a given behaviour (i.e. with more self-determined regulation types found further to the left of Figure 1) and thus the greater the probability that the behaviour will be conducted and maintained (Deci & Ryan, 2012; Gillison, Rouse, Standage, Sebire, & Ryan, 2019; Ng et al., 2012; Ntoumanis et al., 2020). If needs are not satisfied (i.e. dissatisfaction) it is less likely behaviours will be conducted. It is also typical to experience several types of regulation toward one behaviour, and this often occurs, for example, having a mixture of introjected and external regulation to exercise. Although these three needs are theoretically distinct from one another, research has also found that they correlate highly (Standage, Duda, & Ntoumanis, 2005).

Furthermore, an environment can also actively frustrate the basic psychological needs (i.e. need frustration). This situation occurs when someone or something is actively hindering needs (Bartholomew, Ntoumanis, Ryan, & Thøgersen-Ntoumani, 2011a). Need frustration is not equivalent to need dissatisfaction. Vansteenkiste and Ryan (2013) describe a plant lacking the sun, soil, and water, which describes dissatisfaction as it doesn’t have the nutrients to grow and will likely die; whereas, pouring saltwater onto the plant will kill it quicker, which describes frustration. In the context of COPD, dissatisfaction could be a lack of knowledge on what exercises to perform, preventing an individual from satisfying their need for competence. Whereas, dyspnoea could be perceived as actively hindering an individual’s competence to be active and thus be a frustrating stimulus. However, even if an environment is actively frustrating psychological needs, this does not necessarily mean that an individual cannot satisfy them; rather, the individual requires more than the typical amount of effort to achieve this (Bartholomew, Ntoumanis, Ryan, Bosch, & Thøgersen-Ntoumani, 2011b; Warburton, Wang, Bartholomew, Tuff, & Bishop, 2020). For example, an individual can still perceive their competence to be satisfied if they have a lot of knowledge on what exercises to perform, despite dyspnoea actively frustrating their competence needs. General symptoms and acute exacerbations have been described as frustrating stimuli in people with COPD (Toms & Harrison, 2002; Wortz et al., 2012). Studies have also found that the three need frustration variables correlate with one another (Vansteenkiste & Ryan, 2013; Warburton et al., 2020), however, there is a sparsity of research that has investigated the effects of need frustration (Olafsen, Niemiec, Halvari, Deci, & Williams, 2017). This construct has been posited as potentially more important than need satisfaction in predicting behaviour in situations with sub-optimal motivation (Bartholomew et al., 2011b). Wortz et al. (2012) found that people with COPD engaged in self-management behaviours because this enabled them to regain the ability to participate in enjoyable activities. This describes participating in an activity for an inherent reward (regaining abilities) and not for the sheer enjoyment and pleasure of the behaviour (self-management). Thus, this suggests that people are extrinsically motivated to conduct self-management behaviours and represents an activity with ‘sub-optimal motivation’.

Whilst there are still relatively few studies directly applying SDT to populations with COPD (McCullough et al., 2016; Richardson et al., 2014), research has reported the ability of the theory to predict behaviours relevant to COPD self-management, albeit in non-COPD populations (Ng et al., 2012; Ntoumanis et al., 2020; Ryan, Patrick, Deci, & Williams, 2008); including successfully predicting physical activity participation (Brooks et al., 2018; Rahman, Thøgersen-Ntoumani, Thatcher, & Doust, 2011; Rahman, Hudson, Thøgersen-Ntoumani, & Doust, 2015) and smoking cessation (Williams et al., 2006). Multiple studies have also found that higher levels of basic need satisfaction were associated with greater well-being and perceived health (Bartholomew et al., 2011b; Chen, Chang, Tsai, & Hou, 2018; Rahman et al., 2011; Rahman et al., 2015). Whereas, dissatisfaction of the three needs has been correlated to greater depression and anxiety (Uzman, 2014). Thus, as well as predicting self-management, SDT may also be useful for predicting HRQoL for people with COPD.

The relatively few studies that have applied SDT directly to populations with COPD have shown that the basic psychological needs were related to continued self-management processes post-PR (Stewart et al., 2014). Additionally, one study concluded that healthcare providers needed to be cognisant of self-determined regulation for physical activity in this population (Cho, Tung, Lin, Hsu, & Lee, 2017). Furthermore, several studies of PR and self-management in COPD have indicated the importance of satisfying competence and relatedness, although they did not directly relate their findings to SDT (Halding, Wahl, & Heggdal, 2010; Rapport et al., 2014; Turner, Anderson, Wallace, & Kennedy-Williams, 2014). Therefore, although previous literature does suggest that SDT can be applied to populations with COPD, the only studies that explicitly tested the theory in this context, used the model to predict behaviours in individuals who had already completed a PR programme (Cho et al., 2017; Stewart et al., 2014). However, less than 1.5% of those with COPD receive this intervention each year (Yohannes & Connolly, 2004), with some UK waiting-list times extending beyond a year (Baxter et al., 2016). Additionally, 31% of those referred to PR do not even attend the initial assessment (Steiner et al., 2016), illustrating the lack of understanding regarding the motivations of a large proportion of people with moderate-to-severe COPD. Therefore, exploring the application of SDT to predict important indicators of self-management in those who have not attended a PR programme, offers a novel opportunity to understand the regulation of individuals with COPD to self-manage in the period between diagnosis and access to PR.

This study aims to investigate whether SDT constructs can predict regulation to self-manage and HRQoL in people with moderate-to-severe COPD. From the above literature, four hypotheses were formed. 1) That high psychological need satisfaction and low psychological need frustration will predict more autonomous regulation to self-manage. 2) That low psychological need satisfaction and high psychological need frustration will predict more controlled regulation to self-manage. 3) That need frustration will be a stronger predictor of controlled regulation and need satisfaction will be a stronger predictor of autonomous regulation to self-manage. 4) That high psychological need satisfaction and autonomous regulation to self-manage, and low need frustration and controlled regulation, will predict more positive levels of self-management knowledge and HRQoL.

## Materials and methods

### Design

This study used a cross-sectional questionnaire-based design to consider the predictive relations between psychological need satisfaction, psychological need frustration, behaviour regulation to self-manage, HRQoL, and self-management knowledge in a sample with moderate-to-severe COPD. A PR waiting-list population was used for this study because it allowed for consideration of a group with a COPD diagnosis who had received no intensive intervention. Additionally, to be referred to PR, individuals have to be breathless despite optimal medication, meaning that PR is the only treatment left available to help alleviate the condition. Ethical approval was obtained from the NHS Research Ethics Committee (reference number: 16/WA/0068).

### Participants

People with moderate-to-severe COPD on a Hywel Dda University Health Board waiting-list to attend PR during the period October 2016 – May 2018 were invited to participate in this study. To be eligible for referral to PR (and thus on the waiting-list), individuals had to be 40 years or older, have at least 10 pack-years smoking history, breathless despite optimal medication, and post-bronchodilator spirometry of FEV1/FVC ratio less than 70% and FEV_1_ less than 80% predicted. All participants had to be willing and able to give informed consent. No additional exclusion criteria were imposed.

Initial invitation letters were posted to 226 individuals who met the inclusion criteria. A further 115 reminder letters were sent to those who did not respond. A total of 78 people provided written informed consent, representing a 23% response rate for all 341 sent letters. However, six of these people did not return completed questionnaires; therefore, the final sample for this study was 72. The average age was 67.9 (standard deviation [SD] = 9.25). The sample included 35 males and 34 females (3 missing).

### Data collection

Alongside invitation letters, prospective participants were sent a questionnaire booklet consisting of five different measures. If a participant wished to be involved in the study, they were asked to sign the consent form and return this with a completed questionnaire booklet in a prepaid envelope that was provided.

Due to the slight modifications to three of the questionnaires detailed below, the authors’ believed it necessary to measure the internal consistency of the study data. Following published recommendations (Dunn, Baguley, & Brunsden, 2014), to measure the internal consistency, McDonald’s Omega was calculated individually for all modified self-report questionnaires. Following guidelines from published literature, a coefficient between 0.70 and 0.95 was considered acceptable (Bland & Altman, 1997; DeVellis, 2016; Nunnally & Bernstein, 1994). The internal consistency for all questionnaire scales (both individually and aggregated together) were within these guidelines, other than two domains in the need frustration questionnaire that provided an Omega of 0.96. All scores are presented in Table 1.

**Table 1.** Descriptive statistics for questionnaire responders.

	N	Mean (SD)	Questionnaire range	Omega
**HRQoL**	71	27.8 (5.73)	0–40	
**Self-management knowledge**			0–100	
*About COPD*	71	68.1 (21.5)		
*Managing symptoms of COPD*	68	60.4 (18.6)		
*Accessing help and support*	70	47.3 (23.9)		
*Overall*	67	60.5 (17.7)		
**BPN frustration**			4–28	0.96
				0.96
*Autonomy frustration*	69	13.6 (7.47)		
*Competence frustration*	68	16.5 (6.94)		0.94
*Relatedness frustration*	69	9.62 (5.74)		0.93
**BPN satisfaction**			3–18	0.92
*Autonomy satisfaction*	68	13.6 (3.66)		0.89
*Competence satisfaction*	67	10.8 (4.24)		0.95
*Relatedness satisfaction*	64	11.1 (4.02)		0.90
**Regulation to self-manage**			1–5	
*Autonomous regulation*	67	3.57 (0.86)		0.95
*Controlled regulation*	67	2.39 (0.73)		0.88

BPN = basic psychological needs. Omega is provided for questionnaires that were slightly modified only.

The COPD Assessment Test (CAT; Jones et al., 2009) was used to measure participants’ HRQoL. This questionnaire comprises eight questions rated on a six-point Likert Scale. Scores are totalled for the eight questions giving a result between 0 and 40; where, 0 is indicative of low disease impact and 40 is indicative of high disease impact. When up to two scores are missing, these values can be set as the average of the non-missing scores; however, when more than two scores are missing, these values cannot be calculated (Jones et al., 2009).

The Understanding COPD Questionnaire (UCOPD; O'Neill, Cosgrove, MacMahon, McCrum-Gardner, & Bradley, 2012) measures COPD understanding, self-efficacy, and satisfaction, which together will henceforth be referred to as ‘self-management knowledge’. The measure has eighteen items scored using a Likert scale marked 0-10. Questions are divided into three sections: about COPD, managing symptoms of COPD, and accessing help and support. These sections have seven, seven and four items, respectively. A percentage of total self-management knowledge is calculated for each section. An overall score can be calculated for the entire questionnaire. Low percentages are indicative of a participant’s lack of self-management knowledge. O'Neill et al. (2012) offer no guidance regarding missing scores for more than one item or if data is only collected at one time point.

The Psychological Need Thwarting Scale (PNTS; Bartholomew et al., 2011a) involves twelve items rated on a seven-point Likert scale, measuring how an individual feels their basic psychological needs are being actively frustrated. Each basic need has four items. Each item score is aggregated to give a total level of frustration for each of the basic psychological needs. Two modifications were made to the original questionnaire. First, the stem was modified to read as follows ‘Regarding your everyday experiences since being diagnosed with COPD, please indicate on a scale of 1 (disagree) to 7 (agree) how much you can relate to each of the following statements’. Second, individual PNTS items were modified to focus the questionnaire on how needs are being frustrated by COPD. For example, ‘Due to COPD, I feel prevented from making choices regarding the way I live’. These modifications were implemented to allow the questionnaire to measure the role of COPD in frustrating their basic psychological needs.

The Psychological Need Satisfaction in Exercise Scale (PNSE; Wilson, Rodgers, Loitz, & Scime, 2006) consists of eighteen items; however, due to the repetition of items and the total length of all the questionnaires, the number of items were reduced to nine (three items per basic psychological need). The removal of items involved the authors discussing each question and arriving at a consensus as to which to exclude. These items are rated on a six-point Likert Scale with three questions relating to each of the three basic psychological needs. Scores are aggregated for each of the three needs. This questionnaire was modified so that the items were specifically asking about the participants’ basic psychological needs in relation to the self-management of their condition; for example, ‘I feel free to self-manage my condition in my own way’.

The Behavioural Regulation in Exercise Questionnaire-3 (BREQ-3; Wilson, Rogers, Rodgers, & Wild, 2006) involves 24 items rated on a five-point Likert Scale marked 0-4. Each item score is aggregated to give a total score for each of the six different types of motivational regulation (see Figure 1). Due to the sample size, these six regulation types were combined into Autonomous Regulation (Intrinsic, Integrated, and Identified) and Controlled Regulation (Introjected, External, and Amotivation). The questionnaire wording was modified to specifically measure participants’ behavioural motivation to the self-management of their condition, rather than solely exercise; for example, ‘I engage with activities that support the self-management of my symptoms because it’s fun’.

### Analysis

All variables met the parametric assumptions of normality and homogeneity of variance except for self-management knowledge, relatedness frustration, and autonomy satisfaction. This was tested using the Kolmogorov–Smirnov test with the Lilliefors significance correction because of the relatively large sample size in this study making it the most appropriate test. Raw data were not transformed to rectify parametric assumption violations. This decision is supported by previous research which has found that transforming raw data in such situations won’t necessarily affect residuals (Field, 2013).

Path analyses were used to test the four hypotheses using SPSS Amos. The analyses used maximum likelihood and estimated means and intercepts due to small amounts of missing data. Cases were removed list-wise. Chi-square tests compared over- and just-identified models for each analysis. For testing incremental, parsimonious, and absolute fit, the comparative fit index (CFI), the CMIN/DF (minimum discrepancy divided by degrees of freedom), and the root mean squared approximation of error (RMSEA) were used, respectively. These three fit indices are resilient against sample size effects (Hoe, 2008). CFI of 0.90 or greater, CMIN/DF ratio of 3 or less, and RMSEA of 0.05 or less, represent a good fit between the model and the data (Bentler, 1990; Hoe, 2008; Kline, 2015). RMSEA between 0.08 and 0.05 is considered an *acceptable* fit (Awang, 2012).

## Results

Descriptive statistics, including McDonald’s Omega, for all measured variables are displayed in Table 1. It is important to note that higher scores on the HRQoL measure (CAT) are indicative of worse health states; therefore, HRQoL was found to be relatively poor in the people who participated in this study. However, the other descriptive statistics show relatively high scores across the various measures. For example, averages for the satisfaction of all three needs are comparatively high, in addition to relatively high autonomous regulation to self-manage. Similarly, self-management knowledge scores are rather high, although ‘accessing help and support’ was the lowest scored on average. Lastly, competence frustration represents the most inhibited need, which corresponds with previous descriptions of people with COPD (Russell et al., 2018). However, the averages of all three needs shows relatively low levels of frustration.

**Figure 2.**
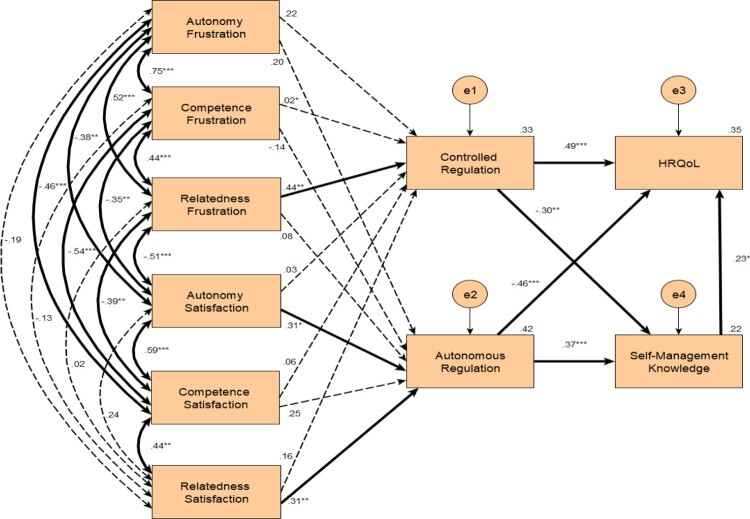
Path Analysis Model of Associations between SDT Constructs, HRQoL, and Self-Management Knowledge. Note: Coefficients presented are standardised linear regression coefficients. Non-statistically significant pathways are denoted with dashed lines. *p < 0.05, **p < 0.01, ***p < 0.001.

Constructs were entered into the model and relations drawn between these constructs, based on the theoretical principles of SDT, as described in the introduction (Ng et al., 2012; Ryan et al., 2008; Ryan & Deci, 2017). To briefly summarise, published research has reported the high correlation between need satisfaction variables (Standage et al., 2005). Likewise, need frustration variables have also been found to be correlated (Vansteenkiste & Ryan, 2013; Warburton et al., 2020). There is a wealth of literature that reports that the basic psychological needs can predict behavioural regulation (Hagger, Chatzisarantis, & Harris, 2006; Ng et al., 2012). Ryan et al. (2008) also describe the relations between SDT constructs, behaviours pertinent to self-management knowledge (e.g. smoking cessation and physical exercise), and HRQoL. Lastly, literature has reported the positive relationship between self-management knowledge and HRQoL (Majothi et al., 2015; Zwerink et al., 2014); therefore, this relationship was also replicated within the model (see Figure 2).

A Chi-square test found no statistically significant differences between the proposed model (i.e. Figure 2) and the just-identified model (χ^2^ (13, N = 72) = 16.7, *p > *0.05). Additionally, CMIN/DF, CFI, and RMSEA were 1.28, 0.93, and 0.06, respectively. Thus, representing an acceptable fit to the data (Awang, 2012; Bentler, 1990; Hoe, 2008; Kline, 2015).

To explore whether this model could be improved, the non-statistically significant pathways (dashed lines in Figure 2) were removed and the analysis conducted again. However, this did not result in a better fitting model (CMIN/DF = 1.29, CFI = 0.97, and RMSEA = 0.06); thus, the original was kept and is discussed further below.

All the relations between the basic psychological needs were in their predicted direction. The three need frustration variables were positively correlated with one another, with autonomy and competence frustration having a particularly strong relationship. Similarly, the satisfaction of the three needs were also positively correlated. Negative correlations were found between need frustration and need satisfaction variables, however, some of these were not statistically significant.

The only statistically significant psychological needs to predict self-management regulation were relatedness frustration, which predicted 33% of the variance for controlled regulation, and autonomy and relatedness satisfaction, which predicted 42% of the variance for autonomous regulation. All associations were positive. Autonomy and competence frustration and competence satisfaction did not directly predict either of the regulation types.

Both controlled and autonomous regulation had strong predictive relations (negative and positive, respectively) with self-management knowledge, accounting for 22% of the variance of this variable. Additionally, controlled and autonomous regulation had strong predictive relations (positive and negative, respectively) with HRQoL. It is important to remember that the CAT (the HRQoL measure) is negatively scored; therefore, the results indicate that more controlled regulation predicts lower HRQoL and more autonomous regulation predicts higher HRQoL. 35% of the variance for HRQoL was accounted for by the variables controlled and autonomous regulation to self-manage and self-management knowledge. Unexpectedly, however, greater self-management knowledge predicted worse HRQoL.

## Discussion

The aim of this study was to explore the ability of SDT to predict regulation to self-manage and HRQoL in a sample of individuals with moderate-to-severe COPD who had not attended a PR programme. Using the path analysis the model showed an acceptable fit to the data; therefore, supporting the acceptance of the four hypotheses. However, none of the psychological need satisfaction variables directly predicted controlled regulation and no psychological need frustration variables directly predicted autonomous regulation; therefore, the first two hypotheses can only be partially accepted.

Increasing self-management knowledge and HRQoL has been identified as crucial to support people with COPD (Kielmann et al., 2010) and, although contrary to previous literature (Gillison et al., 2019; Ntoumanis et al., 2020), the path analysis suggests that controlled and autonomous regulation to self-manage could both be variables to consider in this endeavour. The direct effects of controlled and autonomous regulation to self-manage on HRQoL were especially strong, with 35% of the variance of this variable being explained by the two self-management regulation types and self-management knowledge. The strength of the relations between the SDT variables and health outcomes highlights the importance of targeting motivational factors when developing interventions for people with COPD. This corroborates past literature that identified a lack of motivation as one explanation for self-management behaviours not being conducted (Hillebregt et al., 2016; Russell et al., 2018); however, may also suggest that it is not merely a lack of motivation as a whole, but potentially a lack of one or more of the autonomous self-regulation types or the satisfaction of psychological needs that is responsible (Hagger & Chatzisarantis, 2007; Sheldon et al., 2003). SDT provides clear guidelines for each basic psychological need to allow interventions to be developed based upon its tenants (Hagger & Chatzisarantis, 2015) and a recent study providing descriptions for 21 motivation and behaviour change techniques (MBCTs) has facilitated this ability further (Teixeira et al., 2020). Therefore, by combining the results of this study with the techniques identified, potentially the most important needs for decreasing controlled self-management regulation, and increasing autonomous regulation, self-management knowledge, and HRQoL (i.e. relatedness frustration and autonomy and relatedness satisfaction), may begin to be targeted by future interventions. Meta-analyses conclude that SDT interventions can increase autonomous regulation (Gillison et al., 2019; Ntoumanis et al., 2020), however, a recent review found small and non-significant associations between the same interventions and controlled motivation (Ntoumanis et al., 2020). Nevertheless, the authors reiterate the need for future healthcare studies to investigate how internal and external pressures, and feelings of helplessness (i.e. more controlled forms of motivation), can be influenced (Ntoumanis et al., 2020). This study responds to these suggestions and describes that it may be need frustration that requires further investigation to affect controlled motivation. Although future and larger studies would be needed to replicate the findings of this study, if this is successfully conducted, potential changes to interventions and standard practices may be relatively minor, due to several of the MBCTs being relatively straightforward to apply. For example, for autonomy, providing a meaningful rationale behind a course of action or providing a choice of different treatments can be beneficial. Additionally, for relatedness, providing opportunities for ongoing support, or encouraging questions, can facilitate this need (Teixeira et al., 2020). However, authors do state that there remains uncertainty as to whether MBCTs can be used to reduce the frustration of psychological needs, as most research and expertise focus on the satisfaction of needs instead (Ntoumanis et al., 2020; Olafsen et al., 2017; Teixeira et al., 2020).

This study corroborates findings that there is an extrinsic element to people with COPDs’ motivational regulation to self-manage (Wortz et al., 2012). Previous research has suggested that need frustration may play a larger role in situations where optimal motivation cannot be achieved (i.e. extrinsic motivation; Bartholomew et al., 2011b). However, out of the three psychological needs, only relatedness frustration directly predicted controlled regulation to self-manage. Relatedness, in comparison to the other two needs, was reported to be the least frustrated by COPD (see Table 1); therefore, this indicates that even low levels of relatedness frustration may have a negative impact on self-determined regulation to self-manage. For extrinsically motivated activities, relatedness is posited as the instigating basic psychological need (Deci & Ryan, 2012; Ryan, Patrick, Deci, & Williams, 2007). Therefore, if people with COPD are having their relatedness needs frustrated, this could potentially explain the poor attendance rate seen at programmes such as PR (Steiner et al., 2015, 2016), because individuals may not have the *initial* desire or confidence to attend these group situations where they’ll be required to do physically exertive activities. The finding that the lowest scored section on the self-management knowledge questionnaire (the UCOPD) related to confidence to access help and support further supports this theory and future research may wish to explore this further.

Consequently, people with COPD may need an external agent (i.e. a healthcare intervention) to overcome the *frustration* of their relatedness needs. However, attempting to increase self-management through increased relatedness *satisfaction*, by implementing group-based interventions, may only serve to increase autonomous regulation and leave the negative effects of controlled regulation present. Previous research has found that interventions with strong group-exercise components are associated with increased participant engagement (Baker & Fatoye, 2019; Restrepo et al., 2008). Therefore, further research is needed to understand whether such programmes are working by solely increasing relatedness satisfaction or by additionally reducing relatedness frustration and, through this, controlled regulation to self-manage. This corresponds to suggestions from other authors who also call for more research within a health domain to focus on controlled regulation and need frustration (Ntoumanis et al., 2020; Teixeira et al., 2020). Future research should additionally consider what components are most beneficial at the onset of behaviours in addition to maintaining these longitudinally. Given that 31% of people invited to PR do not attend the initial assessment (Steiner et al., 2016), there are a large number of people with COPD for whom an intervention may be beneficial. PR is effective at improving health outcomes (McCarthy et al., 2015) and is also cost-effective (Chakravorty, Fasakin, Paine, Narasimhaiah, & Austin, 2011; Griffiths, Phillips, Davies, Burr, & Campbell, 2001); therefore, any intervention that is successful in improving PR attendance, is likely to have significant benefits for those with COPD. Thus, by highlighting the different elements that combine to motivate an individual with moderate-to-severe COPD to self-manage their condition, this study provides a useful first-step in informing future research, interventions, and healthcare practice. The need for such interventions cannot be understated, because people on a PR waiting-list have already had their medication optimised and are still breathless. Therefore, if these people do not possess the necessary self-management regulation which motivates them to attend a PR initial assessment, they will miss out on the only disease treatment still available to them and be removed from the waiting-list.

Although there is a wealth of literature relating to how the satisfaction of needs can be facilitated (Deci & Ryan, 2012; Hagger & Chatzisarantis, 2015), more research is still needed to fully investigate the factors that appear to frustrate relatedness needs in people with COPD (Bartholomew et al., 2011b; Olafsen et al., 2017; Teixeira et al., 2020). The relevance of relatedness frustration may be even more apparent given the current COVID-19 pandemic. People with COPD are being advised to remain inside and shield themselves and as a result may feel that their needs for relatedness are being progressively impeded. Given that group-based PR programmes have temporarily been stopped – which represented a key opportunity for people with COPD to meet others with the same condition (Rapport et al., 2014) – relatedness frustration could surpass the low levels observed in this study and thus may severely increase controlled regulation to self-manage and thus reduce the likelihood of self-management behaviours being conducted. Effective self-management has been shown to increase HRQoL and decrease hospitalisations (Benzo et al., 2016; Majothi et al., 2015; Zwerink et al., 2014); therefore, the impact of increased controlled regulation to self-manage may have profound and longitudinal consequences for both people with COPD and healthcare systems. This study therefore supports the need for urgent theory-driven debate regarding behaviour change in people with COPD (Karloh, Sousa Matias, & Fleig Mayer, 2020) with consideration of novel mechanisms to support people remotely.

Corroborating previous research, autonomy and relatedness satisfaction were found to positively predict autonomous regulation (Ryan & Deci, 2017). However, no statistically significant association was found between competence satisfaction and autonomous regulation. These findings suggest the relative importance of the satisfaction of the three needs to support autonomous regulation and highlights the need for healthcare professionals and interventions to provide opportunities for people with COPD to satisfy their autonomy and relatedness. If this is accomplished effectively, autonomous regulation to self-manage, and the subsequent positive effects on self-management knowledge and HRQoL, may be facilitated.

In an unexpected finding, the path analysis found that greater self-management knowledge negatively predicted HRQoL; contradicting previous research (Benzo et al., 2016; Zwerink et al., 2014). If correct, one interpretation could be that participants with greater self-management knowledge were aware of the poor outlook associated with COPD and thus the negative symptoms could have been associated with being cognisant of this potential decline. However, path analyses cannot make causal inferences (Streiner, 2003); therefore, it is also possible that this relationship should be reversed and lower HRQoL predicts higher self-management knowledge. Although previous research has not investigated this relationship, it is conceivable that in instances of high disease-impact (i.e. low HRQoL) more self-management knowledge is needed to cope with the burden caused by COPD symptoms. This is supported by the finding that those who are older – and thus are likely to have more advanced COPD – were more proficient at completing self-management behaviours (Korpershoek et al., 2016). Although future research could investigate this theory further, time and resources would likely be better spent increasing self-management knowledge at the beginning of the COPD pathway, rather than investigating the effects of these skills at the end.

This study found that autonomy, competence, and relatedness frustration correlate with one another, supporting previous research (Vansteenkiste & Ryan, 2013; Warburton et al., 2020). This suggests that even though autonomy and competence frustration did not directly predict controlled regulation to self-manage, if healthcare services do reduce the effect of these variables, self-management could be enhanced through the interaction of other basic psychological needs. Additionally, the need frustration and need satisfaction measures negatively correlated with one another, as expected, apart from several of the pathways connected to the relatedness satisfaction variable, which were not statistically significant. Consequently, healthcare services could employ MBCTs aiming to increase need satisfaction, which have additional positive effects on the frustration of these needs (or vice versa), by either directly decreasing frustration or indirectly through need satisfaction, leading to significant benefits in more self-determined self-management regulation and HRQoL. However, due to the lack of a relationship between the two measures of relatedness, MBCTs designed to increase the satisfaction of this need would be unlikely to simultaneously decrease need frustration. Thus, as described earlier, future research is needed to fully explore the frustration of relatedness and how best to reduce this factor and provide healthcare services with techniques to use with people under their care.

Although this research has begun to increase the knowledge surrounding the role of motivation and regulation to self-manage in people with moderate-to-severe COPD and suggested specific constructs that future interventions could target for the greatest effect, the study does also have its limitations. Firstly, the use of cross-sectional methods has helped to explore an important population; however, this design does limit the ability of the study to understand how the basic psychological needs change over time and the resulting impact this has on long-term controlled and autonomous regulation to self-manage and HRQoL. Additionally, by not employing any intervention, the study also cannot provide evidence on whether a SDT-based intervention would be beneficial within this population; although, given the universal nature of SDT concepts, such as the basic psychological needs (Deci & Ryan, 2012; Hagger & Chatzisarantis, 2015; Ryan & Deci, 2017), there is no evident reason why an intervention wouldn’t be applicable. Nevertheless, future research could employ multiple data collection points whilst implementing a healthcare intervention to overcome these limitations. Such a project should additionally aim to recruit a larger sample size than the current study, to facilitate the generalisation of findings and fully test the factorial validity of the modified questionnaires, which was unable to be conducted in this study. Secondly, this study has highlighted the importance of relatedness frustration; however, the methodological approach of using questionnaire data meant that findings were unable to provide understanding of the factors contributing to participants’ need frustration. Future research using qualitative methodology could provide more in-depth information and fully elucidate what contributes to this construct and further understand its effect on controlled regulation to self-manage and HRQoL in people with COPD. Lastly, although this study implemented several questionnaires to measure the level of self-management knowledge and behavioural regulation, it only considered self-management as an all-encompassing term and did not capture engagement in specific self-management behaviours (e.g. exercise, smoking cessation, attending appointments, etc.). This decision was made partly due to the large number of different self-management behaviours that are relevant to COPD (NICE, 2018), which made it impractical to measure directly, but also due to issues with respondent memory (Del Boca & Noll, 2000), which made it unlikely that participants would be able to accurately answer had they been asked. However, it is likely that individuals may experience differing levels of self-determined motivation to manage different behaviours and, as such, more in-depth research could highlight differing relations between SDT constructs and regulation of specific behaviours enhancing tailored interventions further.

In conclusion, this study suggests that SDT concepts can be applied to a population with moderate-to-severe COPD, who have not attended PR, to predict controlled and autonomous regulation to self-manage, self-management knowledge, and HRQoL. The research begins to address a gap in the literature, where only a limited number of previous studies have applied SDT to people with COPD. The novel application of this theory has allowed for an increased understanding of self-management in people with COPD and begins to provide interesting and unique contributions pertinent to healthcare professionals aiming to increase self-management knowledge and HRQoL. Specifically, this research suggests that although competence needs are the most negatively affected in this population, it is relatedness frustration that more strongly predicts controlled regulation and the negative affects this has on self-management knowledge and HRQoL. Thus, there is a necessity to further explore what aspects of COPD actively frustrate relatedness needs in order to develop effective interventions to promote self-management behaviours, including improved attendance at PR.

## Data Availability

Due to the nature of this research, participants of this study did not agree for their data to be shared publicly, so supporting data is not available.
